# A Delphi Consensus on Tildrakizumab Dosing and Titration in Moderate-to-Severe Plaque Psoriasis

**DOI:** 10.2340/actadv.v106.adv-2026-0593

**Published:** 2026-08-27

**Authors:** Julia-Tatjana Maul, Maria Concetta Fargnoli, Spyridon Gkalpakiotis, Jo Lambert, Andreas Pinter, Ricardo Ruiz-Villaverde, Paul Sator, Thrasyvoulos Tzellos

**Affiliations:** 1 University Hospital Zurich, Zurich, Switzerland; 2 Faculty of Medicine, University of Zurich, Zurich, Switzerland; 3 San Gallicano Dermatological Hospital, IRCCS, Rome, Italy; 4 Department of Dermatovenereology, University Hospital Královské Vinohrady, Prague, Czech Republic; 5 Third Faculty of Medicine, Charles University, Prague, Czech Republic; 6 Department of Dermatology, Ghent University Hospital, Ghent, Belgium; 7 Department of Dermatology, Venereology and Allergology, University Hospital Frankfurt Am Main, Frankfurt am Main, Germany; 8 San Cecilio University Hospital, Granada, Spain; 9 Instituto de Investigación Biosanitaria de Granada (ibs.GRANADA), Granada, Spain; 10 Department of Dermatology, Clinic Hietzing, Vienna, Austria; 11 Department of Dermatology, NLSH Bodø, Bodø, Norway; 12 Department of Clinical Medicine, UiT Arctic University of Norway, Tromsø, Norway

**Keywords:** psoriasis, tildrakizumab, biologic therapy, personalized medicine, Delphi consensus, dose selection

## Abstract

Tildrakizumab is approved for moderate-to-severe plaque psoriasis in 2 doses, 100 mg and 200 mg, with comparable safety profiles. This study aimed to establish pan-European consensus on optimal tildrakizumab dosing and titration using a modified Delphi methodology. A steering group of 8 European dermatologists developed 48 consensus statements addressing tildrakizumab prescribing, dose selection and titration. The statements were incorporated into an online Likert survey and distributed across 9 European countries to experienced dermatologists. Consensus was predefined as ≥75% agreement. Seventy-two dermatologists completed the survey. Consensus was achieved for 35/48 statements (73%). Based on the results, initiation with the 100 mg dose may be considered in patients weighing <90 kg with a body mass index <25, moderate disease severity or who are biologic-naïve. Initiation with the 200 mg dose may be considered in patients weighing ≥90 kg and/or with a body mass index ≥25, severe disease, high-impact area involvement in the context of severe disease, prior interleukin-23p19 inhibitor failure or failure of ≥2 biologics. Up-titration to 200 mg may be considered for suboptimal response, while down-titration to 100 mg may be considered after ≥1 year of sustained remission. Adoption of this guidance may facilitate more consistent, individualized dosing and improved patient outcomes.

**SIGNIFICANCE**

Tildrakizumab is a treatment for moderate-to-severe plaque psoriasis available in 2 doses (100 mg and 200 mg) with similar safety profiles. However, guidance on selecting the most appropriate dose remains controversial, leading to variation in clinical practice. This study established expert consensus across Europe on which patients are best suited to each dose and when to consider dose titration. The findings provide practical, consensus-informed recommendations to support more consistent and individualized treatment decisions and improve patient outcomes.

Tildrakizumab is a humanized monoclonal antibody that selectively targets the p19 subunit of interleukin-23 (IL-23) and is approved for the treatment of moderate-to-severe plaque psoriasis. It is administered subcutaneously at weeks 0 and 4, followed by maintenance dosing every 12 weeks, with 2 available doses of 100 mg and 200 mg (1, 2). In the phase III reSURFACE 1 and reSURFACE 2 trials, both doses showed comparable safety profiles, supporting dosing based on individual patient characteristics (1, 3).

The Summary of Product Characteristics (SmPC) for tildrakizumab recommends a starting dose of 100 mg; however, in patients with high disease burden or body weight ≥90 kg, the 200 mg dose may be considered at the physician’s discretion (4). *Post hoc* analysis and real-world data (RWD) have identified patient subgroups in whom the 200 mg dose may be associated with improved clinical outcomes, including involvement of high-impact areas, prior biologic exposure, obesity and severe disease (5–9). While several national and international psoriasis management guidelines include tildrakizumab among recommended biologic therapies, many do not differentiate between the 2 doses or provide operational guidance on dose selection and titration, contributing to heterogeneity in prescribing practices (10–13).

Local expert opinions and narrative guidance have been published to support tildrakizumab dosing and titration (3, 14, 15). However, substantial variability remains across recommendations, highlighting the need for a harmonized, multinational approach to guide routine clinical practice. Rather than replacing existing international guidelines, which intentionally remain non-prescriptive regarding dosing, this modified Delphi study sought to establish pan-European expert consensus to provide a practical framework that operationalizes personalized dosing and titration of tildrakizumab in adults with moderate-to-severe plaque psoriasis.

## MATERIALS AND METHODS

**Fig. 1.**
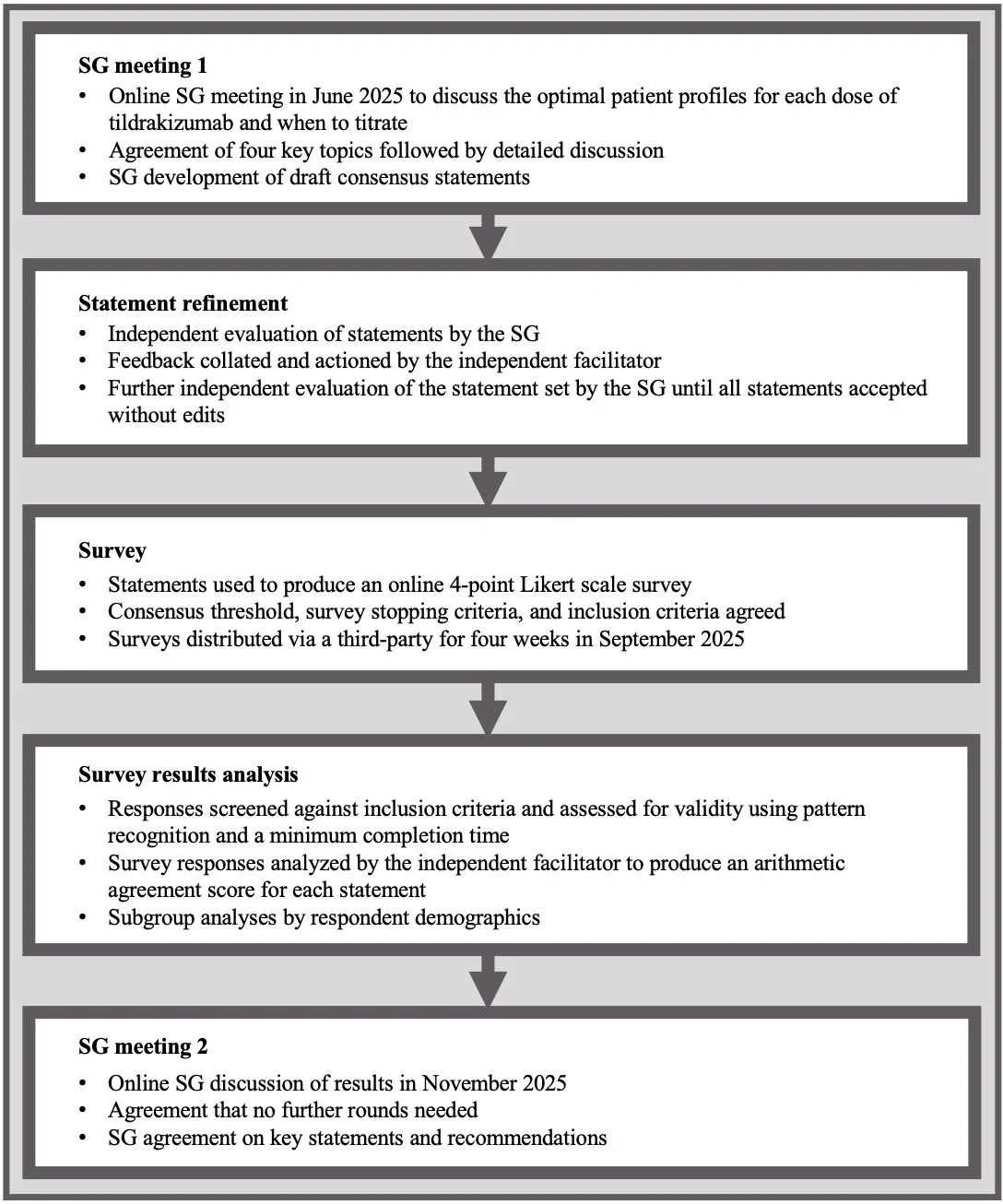
Modified Delphi study design. SG: steering group.

The study followed a modified Delphi methodology (**Fig. 1**), guided by an independent facilitator (Triducive Partners Limited), to systematically capture expert opinion while mitigating the risks of group biases (16). As this was a non-interventional consensus study involving healthcare professionals only, prospective registration was not required, and all reporting adheres to the ACCORD (ACcurate COnsensus Reporting Document) guidelines (17).

In April 2025, a targeted literature review was conducted to inform the aims and study scope and provide context for the first steering group (SG) meeting. The review focused on psoriasis guidelines, predictors of response, and real-world dosing practices. Searches of publicly available databases (PubMed and Google Scholar) were limited to the past 10 years, and search terms included, but were not limited to, “tildrakizumab”, “guideline”, and “dose”. Publications were screened by title and abstract, and supplemented by free-text web searches. Key evidence was narratively summarized. No formal systematic review methodology was applied.

### SG meeting 1

In June 2025, a European SG of 8 dermatologists, selected for their clinical expertise in moderate-to-severe psoriasis management and experience prescribing tildrakizumab, convened virtually. Findings from the literature review informed structured discussion, during which the SG agreed on 4 key topics for consensus development: variations in current practice, ideal patient profiles for tildrakizumab 100 mg and 200 mg and ongoing management. The SG collaboratively developed 48 draft consensus statements.

### Statement refinement

Draft statements were collated by the facilitator and independently reviewed by the SG following the meeting as “accept”, “reword”, or “remove”. The SG were also invited to suggest additional statements. Feedback during this stage was qualitative, consistent with Delphi methodology (18). In round 1 of statement review, 25 statements were accepted without changes, 12 were reworded, 10 were removed and 11 new statements were added. The facilitator incorporated the revisions and circulated the updated statements to the SG for a second independent review. All statements were accepted, resulting in a final set of 48 consensus statements. Fourteen of these statements were designed as paired statements, differing by a single factor (e.g. clinical score, threshold or time frame) with standardized wording, to enable comparison of agreement rates across the options.

### Survey

An online survey was created containing the 48 statements, each presented using a 4-point Likert scale (“strongly disagree”, “tend to disagree”, “tend to agree”, “strongly agree”). Respondents were required to provide qualitative feedback if they disagreed with any statements to explain their reasoning. The survey was distributed online in September 2025 by a third-party provider (M3 Global Research) using convenience sampling and remained open for 4 weeks. Predefined stopping criteria included a target of 72 total responses and fixed quotas per country informed by population size and experience with both doses: Italy, Germany and Spain (*n*=12 respondents per country), and Austria, Belgium, the Czech Republic, France, Sweden and Switzerland (*n*=6 respondents per country). Consensus was defined a priori as ≥75% agreement, consistent with established Delphi methodology standards (19).

Eligible respondents were dermatologists practicing in the predefined countries who were experienced in managing moderate-to-severe plaque psoriasis and individualizing tildrakizumab dosing, as agreed by the SG. Demographic data were captured for subanalyses, including region, years of experience in role, caseload of patients treated with tildrakizumab in the last year and primary institution setting.

The study adhered to the ethical principles outlined in the Declaration of Helsinki and did not require ethics committee approval as it was a non-interventional, opinion-based Delphi process involving healthcare professionals, with no collection of sensitive or participant-identifiable data (20). A statement of consent was included at the start of each survey, and consent was implied by survey completion. All surveys were completed anonymously, and respondents received a nominal fee for participation.

### Survey results analysis

Responses were screened against the inclusion criteria and assessed for validity using pattern recognition and a minimum completion time of 2.5 min. Any respondents not meeting these criteria were excluded. Completed surveys were analyzed by the independent facilitator to produce an arithmetic agreement score for each statement using Microsoft Excel. Agreement was calculated as the proportion of respondents selecting “tend to agree” or “strongly agree” for each statement, divided by the total number of responses. Percentages were rounded to the nearest whole number prior to assessment against the predefined ≥75% consensus threshold.

### SG meeting 2

The SG reconvened online in November 2025 to review and interpret the survey results. Following evaluation of agreement distributions and qualitative feedback, the group determined that additional Delphi rounds were unlikely to meaningfully change the consensus outcomes; therefore, no further rounds were conducted. Key statements were identified for each topic, and draft recommendations were formulated based on the consensus findings. The recommendations were independently reviewed and refined by each SG member during the manuscript development process.

## RESULTS

In total, 72 dermatologists practicing across the predefined European countries completed the survey (Fig. S1). Most respondents had ≥5 years of experience in their role (90%), and 60% had treated ≥5 patients with tildrakizumab in the past year (Figs S2 and S3). Practice settings were primarily academic/university hospitals (46%), followed by public (26%) and private hospitals (20%) (Fig. S4).

**Fig. 2.**
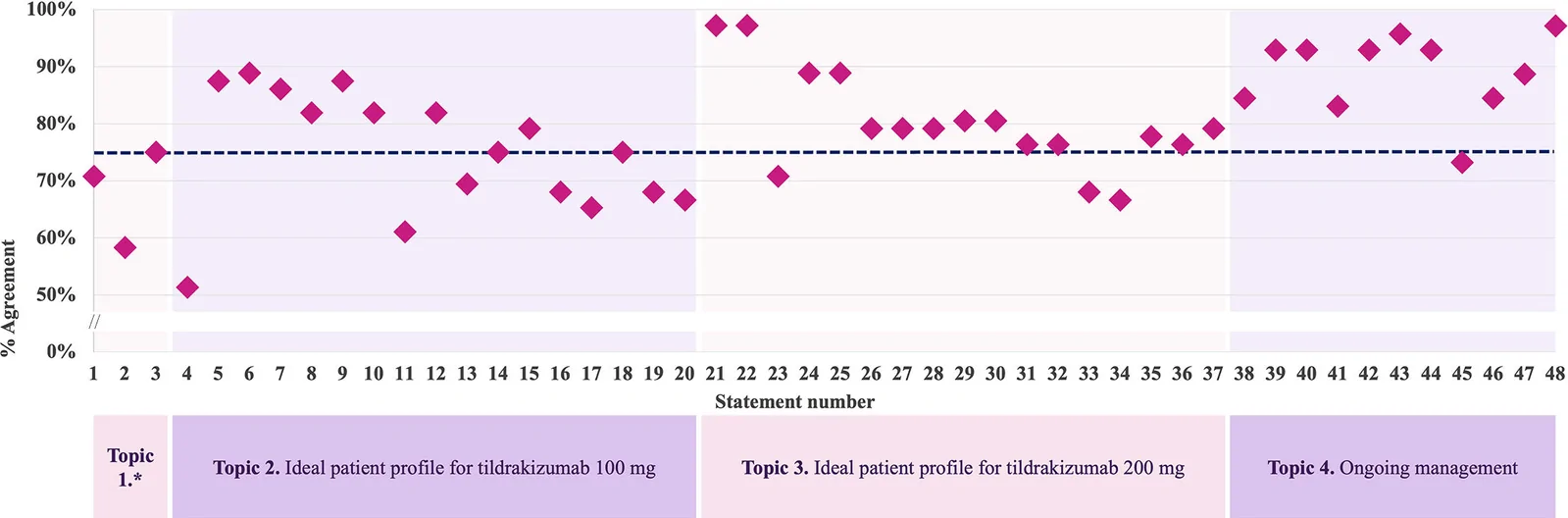
Consensus agreement levels by statement. The threshold for consensus is depicted by the dark blue line (75%). *Variations in current practice.

Consensus was achieved for 35 of 48 statements (73%) (**Table I**; **Fig. 2**). The lowest levels of agreement were observed in Topic 2, assessing the ideal patient profile for the 100 mg dose of tildrakizumab. Subanalyses exploring consensus across demographic subgroups revealed substantial heterogeneity in levels of agreement across all topics, demonstrating the need for this Delphi study (Figs S5–S8).

**Table I.** Defined consensus statements and corresponding levels of agreement. Some statements use standardized wording to allow comparison of agreement rates across alternative clinical scores, thresholds or time frames

No:	Statement:	Agree-ment Score^a^ (***n***=72)
**Topic 1. Variations in current practice**		
**1**.	International, national and local guidelines do not distinguish between the 100 mg and 200 mg doses of tildrakizumab, leaving dosing decisions to the discretion of the prescriber	**71%**
**2**.	Similar safety profiles of both doses of tildrakizumab lead some clinicians to unnecessarily initiate treatment at the higher dose	**58%**
**3**.	The lack of specific training on tildrakizumab dose optimization contributes to heterogeneous prescribing patterns across different levels of care	**75%**
**Topic 2. Ideal patient profile for tildrakizumab 100 mg**		
**4**.	All patients should be initiated on the 100 mg dose	**51%**
**5**.	Patients with a body weight of <90 kg should be considered for the 100 mg dose	**88%**
**6**.	Patients with a body mass index (BMI) <25 should be considered for the 100 mg dose	**89%**
**7**.	Frail patients should be considered for the 100 mg dose	**86%**
**8**.	Patients aged ≥65 years should be considered for the 100 mg dose	**82%**
**9**.	Patients with an absolute PASI of >5 and <10 should be considered for the 100 mg dose	**88%**
**10**.	Patients with an absolute PASI of >5 and <16 should be considered for the 100 mg dose	**82%**
**11**.	Patients with an absolute PASI of >5 and <20 should be considered for the 100 mg dose	**61%**
**12**.	Patients with a DLQI score >5 and <10 should be considered for the 100 mg dose	**82%**
**13**.	Patients who have no involvement of high-impact areas (e.g. genital or palmoplantar regions) should be considered for the 100 mg dose	**69%**
**14**.	Patients with a history of cancer or undergoing cancer treatment should be considered for the 100 mg dose	**75%**
**15**.	Bio-naïve patients should be considered for the 100 mg dose	**79%**
**16**.	Patients who have failed previous tumour necrosis factor-alpha (TNF-α) therapy should be considered for the 100 mg dose	**68%**
**17**.	Patients who have failed previous interleukin-17 (IL-17) therapy should be considered for the 100 mg dose	**65%**
**18**.	Patients naïve for IL-23 inhibitors should be considered for the 100 mg dose	**75%**
**19**.	Patients who have failed only 1 previous biologic and are naïve for IL-23 inhibitors should be considered for the 100 mg dose	**68%**
**20**.	Patients who have failed only 2 previous biologics and are naïve for IL-23 inhibitors should be considered for the 100 mg dose	**67%**
**Topic 3. Ideal patient profile for tildrakizumab 200 mg**		
**21**.	Patients with a body weight of ≥90 kg should be considered for the 200 mg dose	**97%**
**22**.	Patients with a BMI of ≥25 should be considered for the 200 mg dose	**97%**
**23**.	Patients with an absolute PASI of ≥10 should be considered for the 200 mg dose	**71%**
**24**.	Patients with an absolute PASI ≥16 should be considered for the 200 mg dose	**89%**
**25**.	Patients with an absolute PASI of ≥20 should be considered for the 200 mg dose	**89%**
**26**.	Patients with a DLQI ≥10 should be considered for the 200 mg dose	**79%**
**27**.	Patients experiencing significant itch, pain and discomfort should be considered for the 200 mg dose	**79%**
**28**.	Patients with genital involvement should be considered for the 200 mg dose	**79%**
**29**.	Patients with palmoplantar involvement should be considered for the 200 mg dose	**81%**
**30**.	Patients with severe scalp involvement (e.g. a Static Physician’s Global Assessment score of 4) should be considered for the 200 mg dose	**81%**
**31**.	Patients with nail involvement should be considered for the 200 mg dose	**76%**
**32**.	Patients who have a history of frequent or multiple flares, loss of absolute PASI ≤2, and no stable remission should be considered for the 200 mg dose	**76%**
**33**.	Patients with cardiometabolic syndrome (a comorbidity of psoriasis) should be considered for the 200 mg dose	**68%**
**34**.	Patients with a long disease duration of >10 years should be considered for the 200 mg dose	**67%**
**35**.	Patients with previous failure on another IL-23 inhibitor should be considered for the 200 mg dose	**78%**
**36**.	Patients who have failed 2 or more biologics, regardless of class, should be considered for the 200 mg dose	**76%**
**37**.	Patients who have failed 3 or more biologics, regardless of class, should be considered for the 200 mg dose	**79%**
**Topic 4. Ongoing management**		
**38**.	After 12–16 weeks of treatment with the 100 mg dose, if a patient demonstrates a partial response (absolute PASI score ≥2), up-titration to the 200 mg dose should be considered, with re-evaluation at week 28	**85%**
**39**.	After 24–28 weeks of treatment with the 100 mg dose, if a patient demonstrates a partial response (absolute PASI score ≥2), up-titration to the 200 mg dose should be considered, with re-evaluation at week 40	**93%**
**40**.	Patients who experience an end-of-dose effect at any given time while receiving the 100 mg dose should be considered for up-titration to the 200 mg dose	**93%**
**41**.	Residual changes in the nails, even in patients achieving complete skin clearance (PASI 100), should prompt consideration of up-titration to the 200 mg dose	**83%**
**42**.	If a patient reports unsatisfactory symptom relief and/or an itch NRS score >4, up-titration to the 200 mg dose should be considered	**93%**
**43**.	Patients experiencing frequent infections while on the 200 mg dose should be considered for down-titration to the 100 mg dose	**96%**
**44**.	Patients who have achieved complete remission (PASI 100) for ≥1 year on the 200 mg dose should be considered for down-titration to the 100 mg dose	**93%**
**45**.	Patients who have achieved an absolute PASI of ≤2 at week 16 should be considered for down-titration to the 100 mg dose	**73%**
**46**.	Patients who have achieved an absolute PASI of ≤2 at week 28 should be considered for down-titration to the 100 mg dose	**85%**
**47**.	Patients on the 200 mg dose who have minimal or no itching or pain, no active flares or rapid worsening of plaques should be considered for down-titration to the 100 mg dose	**89%**
**48**.	A combined evaluation using objective tools (PASI, DLQI, itch NRS) and subjective patient-reported outcomes should be central to dose adjustment decisions	**97%**

^a^Percentages were rounded to the nearest whole number prior to assessing whether the threshold for consensus was met.

BMI: body mass index; DLQI: Dermatology Life Quality Index; IL-17: interleukin-17; IL-23: interleukin-23; NRS: numeric rating scale; PASI: Psoriasis Area and Severity Index; TNF-α: tumour necrosis factor alpha.

**Fig. 3.**
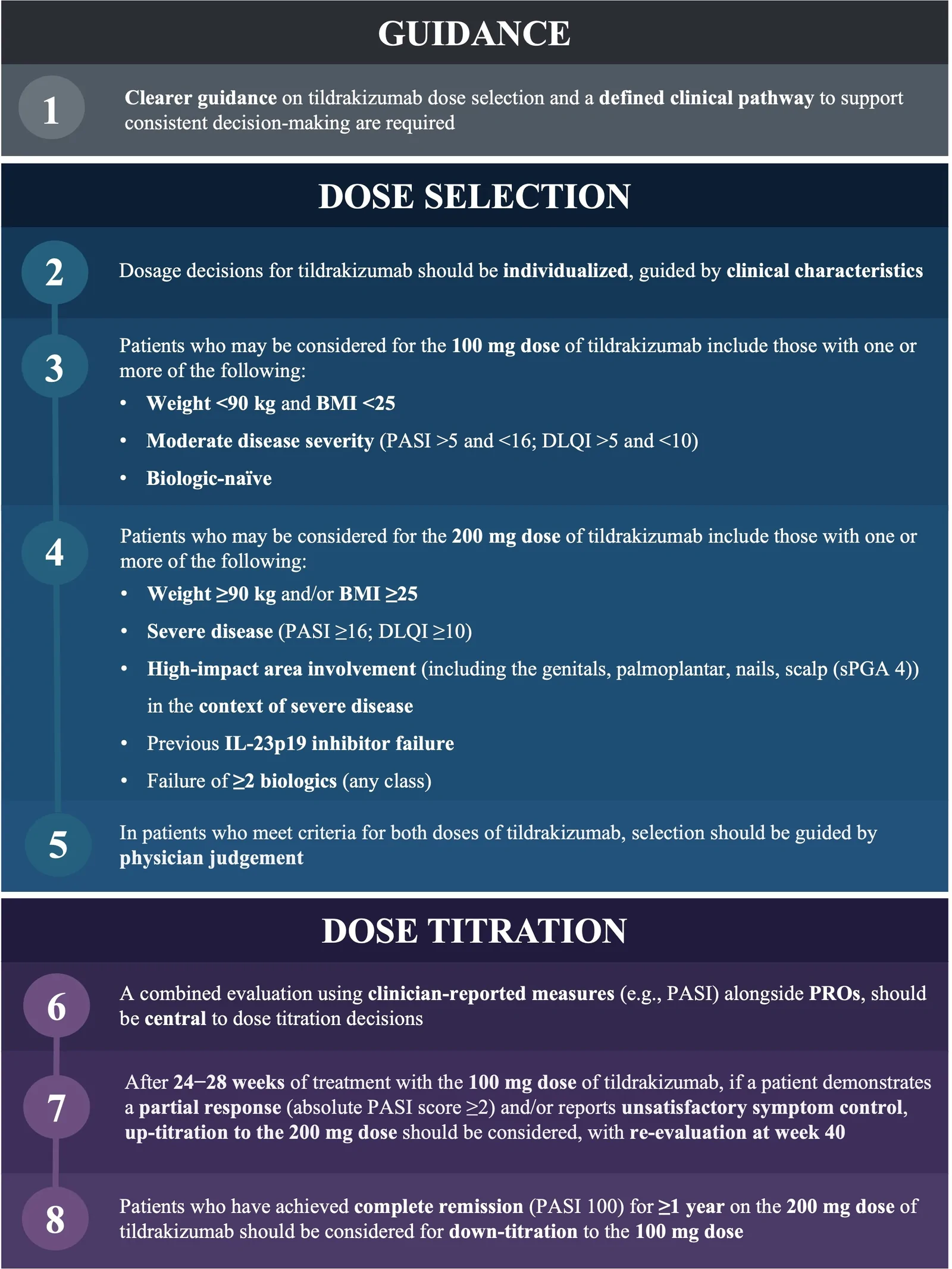
Consensus recommendations based on the Delphi results. BMI: body mass index; PASI: Psoriasis Area and Severity Index; DLQI: Dermatology Life Quality Index; IL-23: interleukin-23; sPGA: static Physician’s Global Assessment; PROs: patient-reported outcomes.

Based on the Delphi findings, the SG developed 8 key recommendations to support clinical decision-making surrounding tildrakizumab dosing and titration (**Fig. 3**).

## DISCUSSION

### Topic 1: Variations in current practice

Uncertainty surrounding optimal tildrakizumab dose selection contributes to inconsistent prescribing practices, highlighting the need for clearer guidance to support decision-making. Consensus on Statement [S] 3 highlights that a lack of specific training on dose optimization is a key driver of this variability (Table I). This variability is further illustrated by divergent views on initial dose selection, where over half of respondents indicated that similar safety profiles between doses may lead to unnecessary initiation at the higher dose (S2, 58%). Together, these findings highlight the need for clearer guidance on tildrakizumab dose selection along with a defined clinical pathway to support consistent decision-making.

### Topics 2 & 3: Ideal patient profiles for tildrakizumab 100 mg and 200 mg

In line with the SmPC, 100 mg should generally be considered the preferred initial dose (4). This preserves the 200 mg dose as an option for up-titration in cases of inadequate or loss of response, potentially avoiding premature switching to alternative biologic therapies and supporting treatment durability. However, initiation with 200 mg should be considered in patients with specific clinical factors to maximize the likelihood of achieving optimal disease control (4–9).

To support individualized tildrakizumab dose selection, a decision-support framework was developed from the Delphi findings (**Table II**). Physicians should consider the clinical factors listed in the framework and assess whether, taken together, they support initiation of the 100 mg or 200 mg dose. In patients who meet criteria for both doses, selection should be guided by physician judgement. This consensus–based framework is intended to support, not replace, individualized clinical judgement and should not be interpreted as a prescriptive guideline. The rationale underlying each factor is discussed in detail below.

**Table II.** Decision-support framework for tildrakizumab dose selection in moderate-to-severe plaque psoriasis

Clinical factors*	Dose (mg)	
	100	200
**Body weight and body mass index**		
Weight <90 kg and body mass index <25		
Weight ≥90 kg and/or body mass index ≥25		
**Disease severity**		
Moderate disease (PASI >5 and <16; DLQI >5 and <10)		
Severe disease (PASI ≥16; DLQ I≥10)		
**Area affected**		
High-impact area involvement (genitals, palmoplantar, nails, scalp [sPGA 4]) in the context of severe disease		
**Treatment history**		
Biologic-naïve		
Previous IL-23p19 inhibitor failure		
Failure of ≥2 biologics (any class)		

Green shading indicates factors supporting dose selection; blank cells indicate limited or no specific support. This consensus–based framework is intended to support, not replace, individualized clinical judgement and should not be interpreted as a prescriptive guideline.

*For patients who meet criteria for both doses, physician judgement should guide selection.

DLQI: Dermatology Life Quality Index; IL-23: interleukin-23; PASI: Psoriasis Area and Severity Index; sPGA: Static Physician’s Global Assessment.

### Body weight and body mass index

There was strong consensus that individuals with body weight <90 kg and body mass index (BMI) <25 should be considered for the 100 mg dose (S5, 88%; S6, 89%), whereas those with body weight ≥90 kg and/or BMI ≥25 should be considered for the 200 mg dose (S21, 97%; S22, 97%). Pooled analyses from 3 randomized controlled trials (RCTs) demonstrated improved response rates with the 200 mg dose in patients with a body weight >90 kg at week 12 (21), and better, although non-significant, Psoriasis Area and Severity Index (PASI) <3 responses at week 28 compared with the 100 mg dose (22). RWD further support these findings, with greater effectiveness observed for the 200 mg dose at week 16 in patients with body weight ≥90 kg (8, 23).

BMI should be considered alongside body weight when determining the tildrakizumab dose, as these measures may not always align; for example, some individuals may weigh <90 kg but have a BMI ≥25, which represents the conventional threshold for the overweight category. High BMI has been associated with reduced clinical responses to biologic therapies in patients with psoriasis, supporting consideration of BMI in dose selection (24). RWD indicates that patients with BMI ≥25 treated with tildrakizumab 200 mg are more likely to achieve low disease activity (PASI ≤5) at week 16 than those with a BMI <25 (5). Consistent with these findings, this consensus supports consideration of the 200 mg dose in patients with a BMI ≥25 (S22, 97%). Overall, body weight and BMI were strongly supported as complementary measures to guide individualized dose selection.

### Disease severity (PASI and Dermatology Life Quality Index)

Patients with moderate disease severity (PASI >5 and <16; Dermatology Life Quality Index [DLQI] >5 and <10) should be considered for the 100 mg dose, whereas those with severe disease (PASI ≥16; DLQI ≥10) should be considered for the 200 mg dose. Statements 9–12 and 23–26 demonstrated consistent alignment on PASI and DLQI thresholds supporting dose differentiation (Table I). This aligns with evidence that shows greater effectiveness with tildrakizumab 200 mg at week 16 in patients with severe disease (PASI ≥16) compared with 100 mg (8, 23). Pooled analyses from the reSURFACE studies and a Phase II study also demonstrated higher response rates to the 200 mg dose in patients with higher baseline disease severity (PASI >20) (59.4%) than to the 100 mg dose (48.6%), supporting enhanced benefit in patients with severe disease (21). Accordingly, this consensus strongly supports disease severity, assessed using a multidimensional approach incorporating PASI and DLQI rather than relying on either measure alone, as an important determinant of dose selection.

### Area of involvement

Patients with high-impact area involvement in the context of severe disease should be considered for the 200 mg dose (S28-31, 76–81%). Both doses of tildrakizumab have demonstrated efficacy in patients with involvement of high-impact areas, including the genitals, palmoplantar surfaces, nails, and scalp (8, 25–28). However, RWD indicates that among patients with involvement of at least one high-impact area, tildrakizumab 200 mg was associated with higher PASI 90 and PASI 100 response rates than 100 mg at week 16, with statistically significant differences maintained through 1 year of follow-up (8). Moreover, patients with severe disease (PASI ≥16) and high-impact area involvement have been shown to achieve higher PASI 90 and PASI 100 response rates and lower residual disease (PASI ≤2) with the 200 mg dose compared with the 100 mg dose at week 16 (23). Therefore, this consensus supports considering area of involvement in the context of severe disease when selecting the tildrakizumab dose.

### Biologic treatment history

Patients who are biologic-naïve should be considered for the 100 mg dose of tildrakizumab (S15, 79%). This aligns with evidence from pooled phase II and III RCTs showing that at week 12, PASI 90, PASI 100, and Physician’s Global Assessment (PGA) responses to tildrakizumab 100 mg were generally higher among patients naïve to biologics for psoriasis (21). RWD further supports this finding, where a greater proportion of patients naïve to biologics achieved better PASI responses at week 24 compared with those previously treated with biologics (29).

In contrast, patients with prior IL-23p19 inhibitor failure or failure of ≥2 biologics (any class) should be considered for the 200 mg dose of tildrakizumab (S35, 78%; S36, 76%). Although direct comparative data specific to prior IL-23p19 inhibitor failure are limited, real-world reports describe clinical benefit with tildrakizumab 200 mg in patients previously treated with other IL-23p19 inhibitors (7). More broadly, evidence for patients who are bio-experienced shows higher PASI 90 and PASI 100 response rates with the 200 mg dose compared with the 100 mg dose (8), and RWD indicates higher response rates when tildrakizumab is used first- or second-line compared with later-line use, with response probability decreasing with each additional treatment (2).

However, several studies have reported no clear association between prior biologic exposure and treatment response, highlighting uncertainty regarding its value as an independent predictor of response (30–33). Treatment history should therefore be considered alongside the other clinical factors identified in this consensus when selecting the tildrakizumab dose (Table II).

### Age, frailty, comorbidities, and disease duration

Although the statements assessing age and frailty in considering the 100 mg dose achieved consensus (S7, 86%; S8, 82%), the SG agreed that these factors alone should not determine dose selection. This is supported by evidence demonstrating comparable long-term PASI and DLQI responses and similar safety and tolerability profiles in younger and older patients (<65 and ≥65 years), irrespective of dose, despite a higher burden of comorbidities and polypharmacy in older patients (34).

In addition, S33 (68%) proposing cardiometabolic syndrome as an indication for the 200 mg dose failed to reach consensus, aligning with evidence showing comparable efficacy, durability of response and safety between the 100 mg and 200 mg doses in patients with and without metabolic syndrome (35). Similarly, S34 (67%) failed to reach consensus, consistent with evidence showing no significant impact of disease duration on tildrakizumab response (36). Accordingly, these factors were not included in the dosing decision-support framework (Table II), as other clinical characteristics (as discussed above) were considered more relevant to dose selection.

### Topic 4: Ongoing management

Psoriasis can have a substantial impact on patients’ quality of life, affecting physical, emotional and psychosocial functioning (37). Evidence shows that clinician-reported outcomes, such as PASI, do not consistently correlate with patient-reported outcomes (PROs). Therefore, reliance on clinician-reported measures alone may provide an incomplete assessment of disease severity and lead to under- or overtreatment (38). Accordingly, a combined evaluation using clinician-reported measures (e.g. PASI) alongside PROs assessing symptoms, such as the itch numeric rating scale (NRS), should be central to dose titration decisions (S48, 97%).

Based on the consensus findings, a decision-support framework was developed to guide tildrakizumab dose titration according to treatment response and timing (**Table III**).

**Table III.** Decision-support framework for tildrakizumab dose titration in moderate-to-severe plaque psoriasis

Current dose	Assessment timepoint	Clinical response	Recommended action	Re-evaluation
100 mg	Week 24–28	Partial response (absolute PASI ≥2) and/or patient-reported unsatisfactory symptom control	Consider up-titration to 200 mg	Week 40
200 mg	≥1 year	Complete remission (PASI 100)	Consider down-titration to 100 mg	Routine follow-up

This consensus–based framework is intended to support, not replace, individualized clinical judgement and should not be interpreted as a prescriptive guideline.

PASI: Psoriasis Area and Severity Index.

### Up-titration

Current treatment guidelines define clinically acceptable treatment goals in psoriasis as achieving an absolute PASI score of ≤3 or ≤2, alongside minimizing the impact of disease on quality of life (39, 40). In line with these targets, up-titration to the 200 mg dose was supported for patients receiving tildrakizumab 100 mg who, after 24–28 weeks of treatment, demonstrate a partial response (absolute PASI ≥2) and/or report unsatisfactory symptom control, with re-evaluation at week 40 (S39, 93%; S42, 93%). Evidence indicates that clinical responses to both tildrakizumab doses continued to improve beyond week 12, with maximal efficacy observed between weeks 22–28 (1, 21). This supports the 24–28 week assessment window recommended by this consensus, thereby avoiding premature dose up-titration in patients who may achieve optimal responses with continued initial dosing.

### Down-titration

While tildrakizumab-specific data on dose down-titration remain limited, this consensus recommends considering down-titration from 200 mg to 100 mg in patients who have maintained complete remission (PASI 100) for ≥1 year (S44, 93%). Down-titration reduces cumulative drug exposure and may support more sustainable long-term management; should disease activity recur, up-titration to the original dose can be considered without the need to switch biologics.

### Strengths and limitations

Strengths of this study include 72 responses from experienced dermatologists, with broad representation across 9 European countries. Response validity was supported by pattern analysis, completion time and agreement consistency, and the use of a 4-point Likert scale reduced neutral response bias. Although the study was industry-funded, safeguards were implemented to minimize potential sponsor influence, including independent survey administration, anonymous participation, external data analysis and interpretation of findings exclusively by the SG. Limitations included insufficient specification of biologic failure and undefined severity thresholds for high-impact areas. Convenience sampling and representation across 9 European countries may limit generalizability and applicability of the findings beyond the represented healthcare settings. In addition, the recommendations are based on consensus findings and require validation to confirm their applicability in routine clinical practice.

### Conclusion

This modified Delphi study achieved pan-European consensus on optimal tildrakizumab dosage and titration for adults with moderate-to-severe plaque psoriasis among dermatologists. The findings highlight substantial variation in current practice and address an unmet need for clearer, standardized recommendations. The consensus recommendations and decision-support frameworks promote a personalized approach to care, integrating clinical characteristics and patient-reported outcomes to guide dose initiation and titration. Their implementation may improve consistency in clinical decision-making and optimize patient outcomes. Future studies are required to validate the consensus outputs and assess their impact on long-term treatment outcomes.

## Data Availability

All data generated or analyzed during this study are included in this published article and the supplementary information files.
