# Supplementary material for: A Delphi Consensus on Tildrakizumab Dosing and Titration in Moderate-to-Severe Plaque Psoriasis
Source: Acta Derm Venereol. 2026 Aug 27;106:0593. doi: 10.2340/actadv.v106.adv-2026-0593 (PMC13520585; doi:10.2340/actadv.v106.adv-2026-0593)
Supplement: Supplementary file 1 [file ActaDv-106-0593-s0001.pdf]

SUPPLEMENTAL MATERIAL

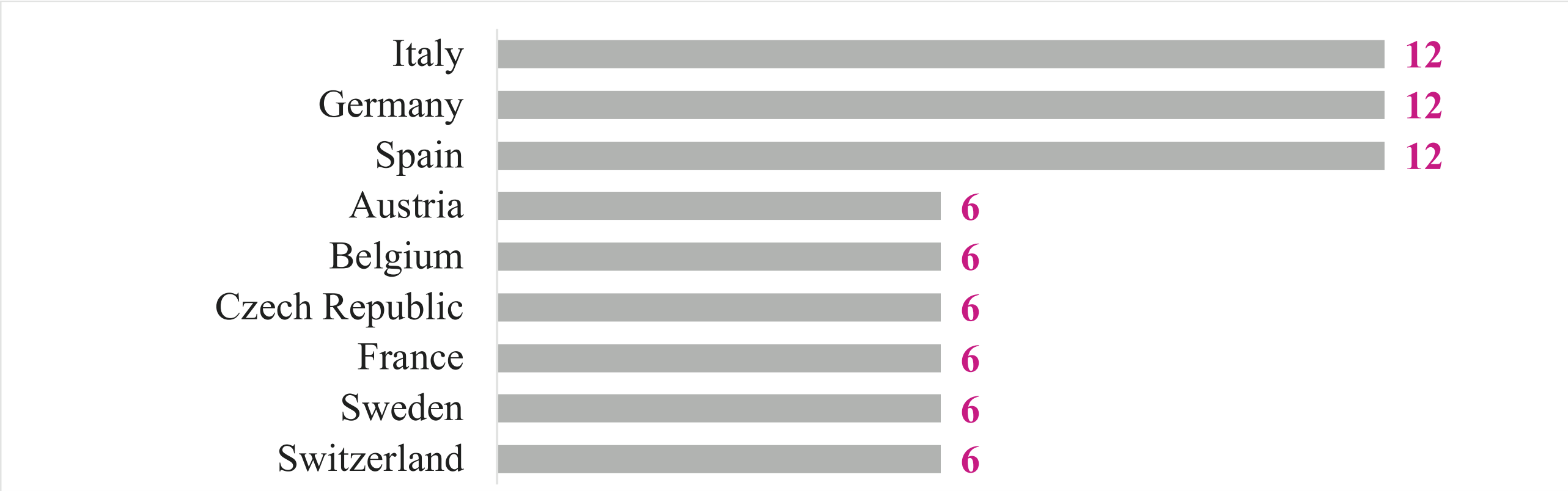

Figure S1. Number of respondents per country.

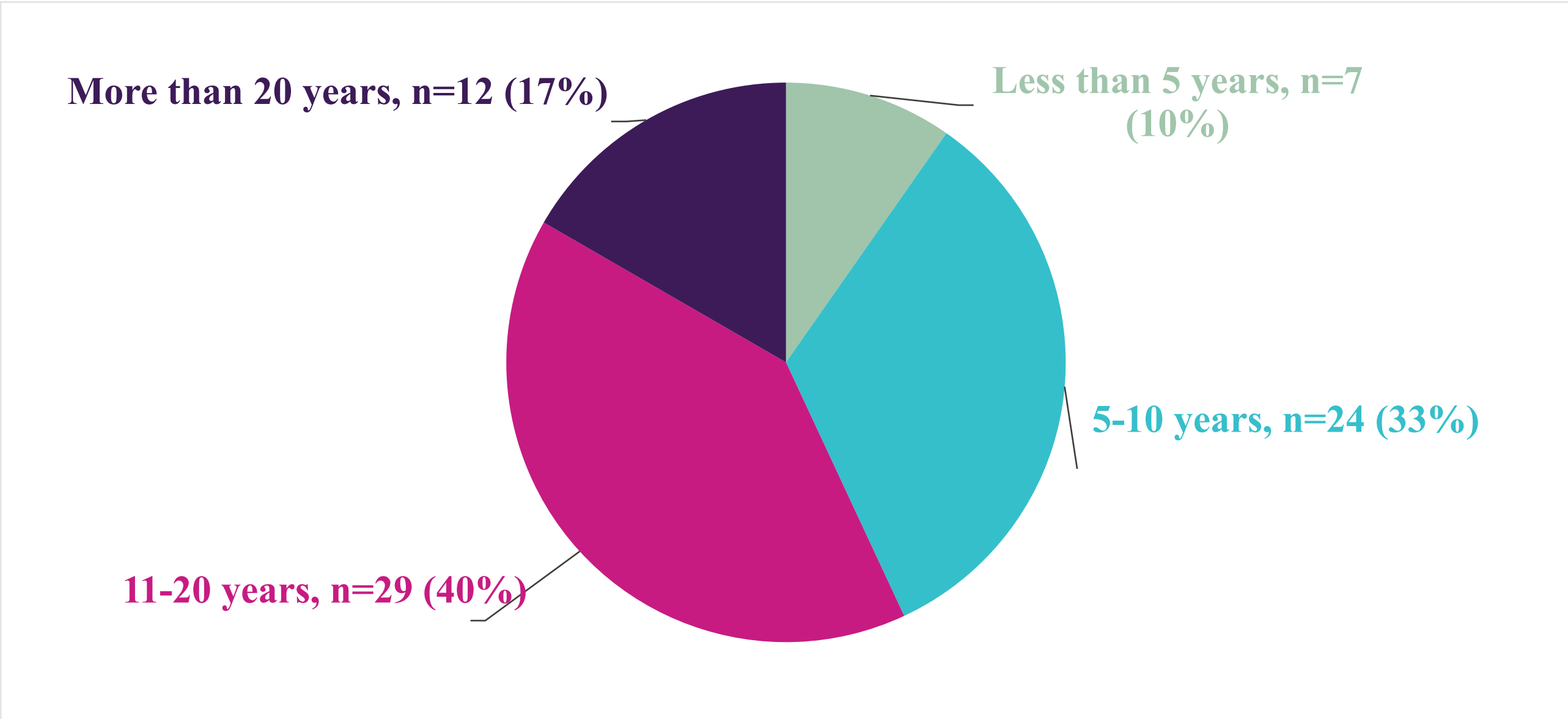

Figure S2. Number of respondents by years of experience in role.

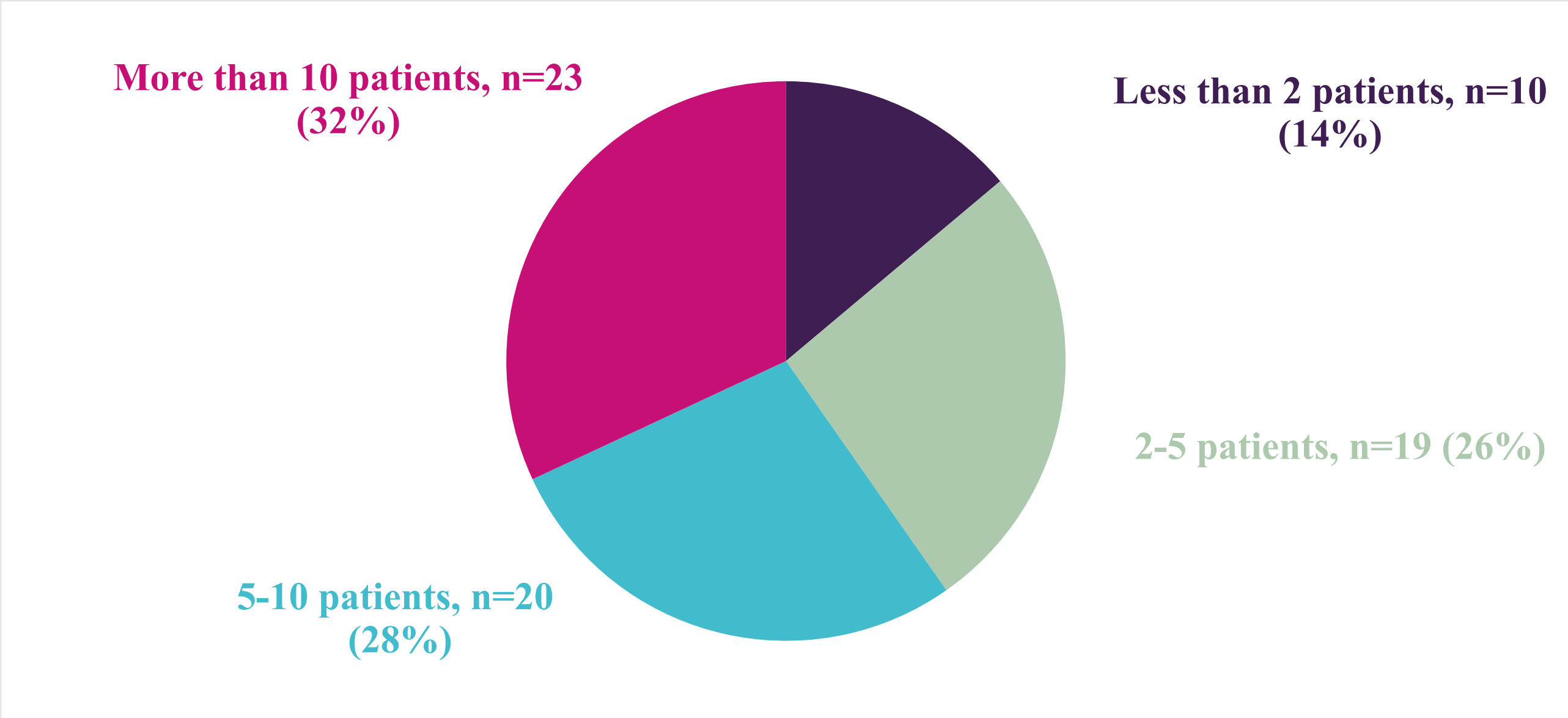

Figure S3. Number of respondents by caseload of patients treated with tildrakizumab in the last year.

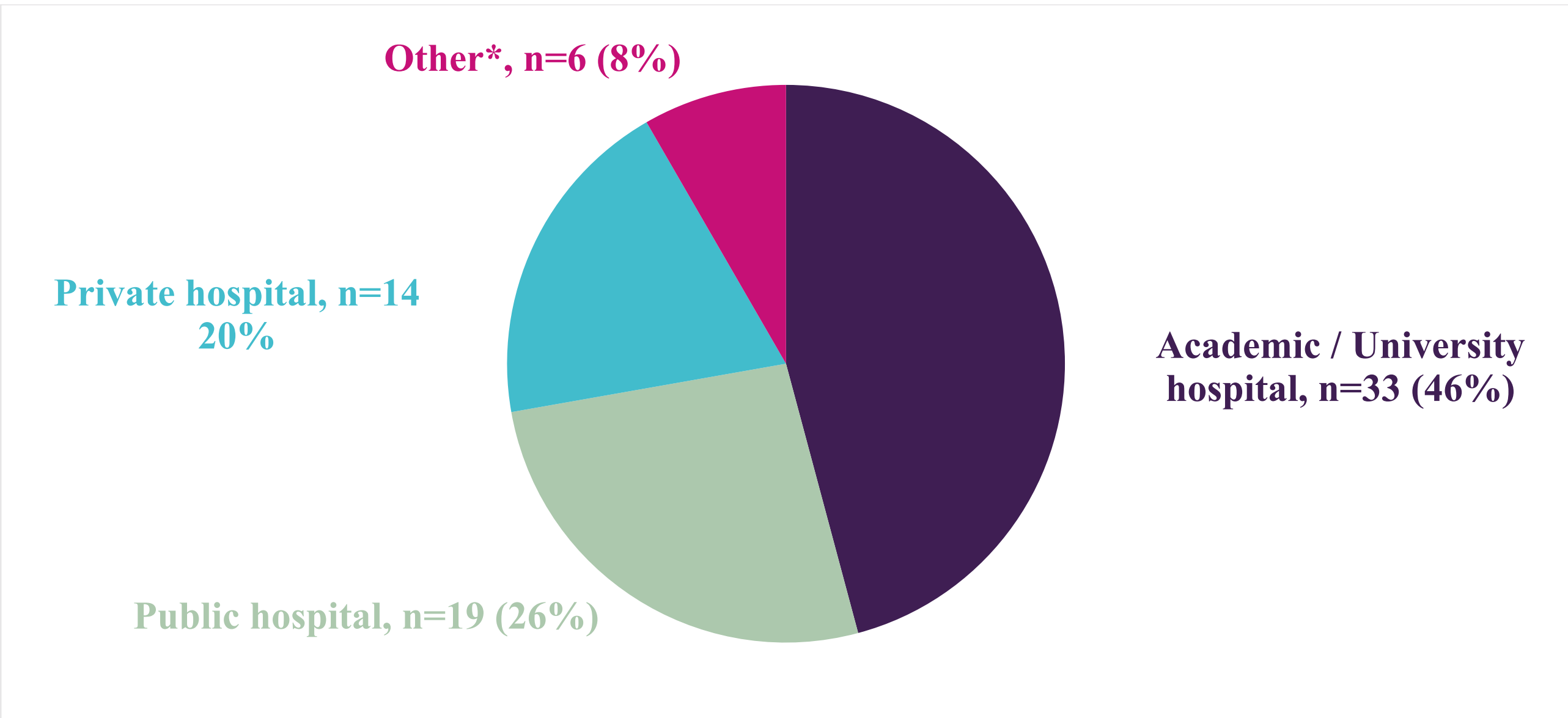

Figure S4. Number of respondents by primary affiliated institution. *Other includes Dermatology practice (n=2), Medical Care Centre (n=1), Group practice (n=1), Dermatology group clinic (n=1), and Office-based private practice (n=1).

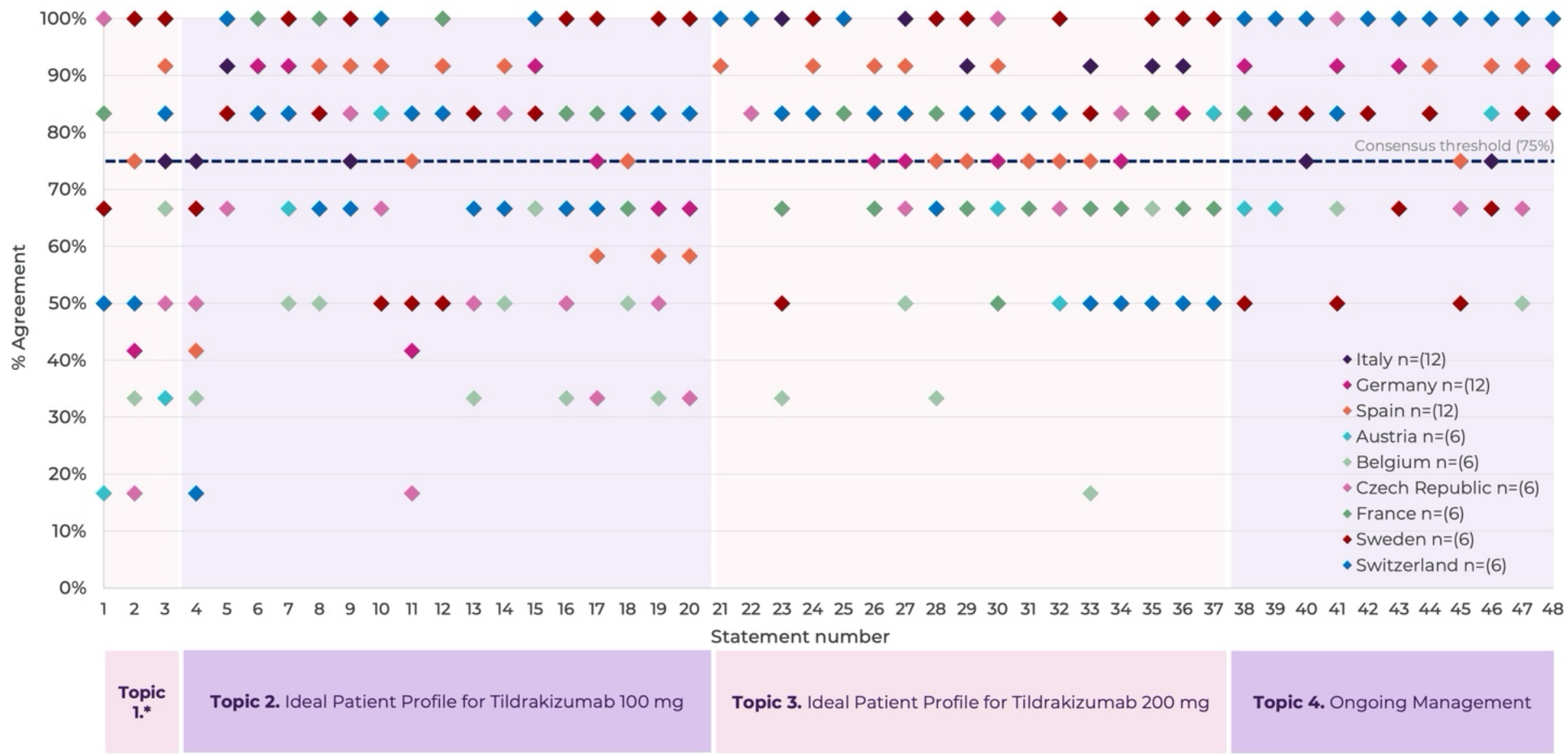

Figure S5. Sub-analysis of responses by country. *Variations in current practice.

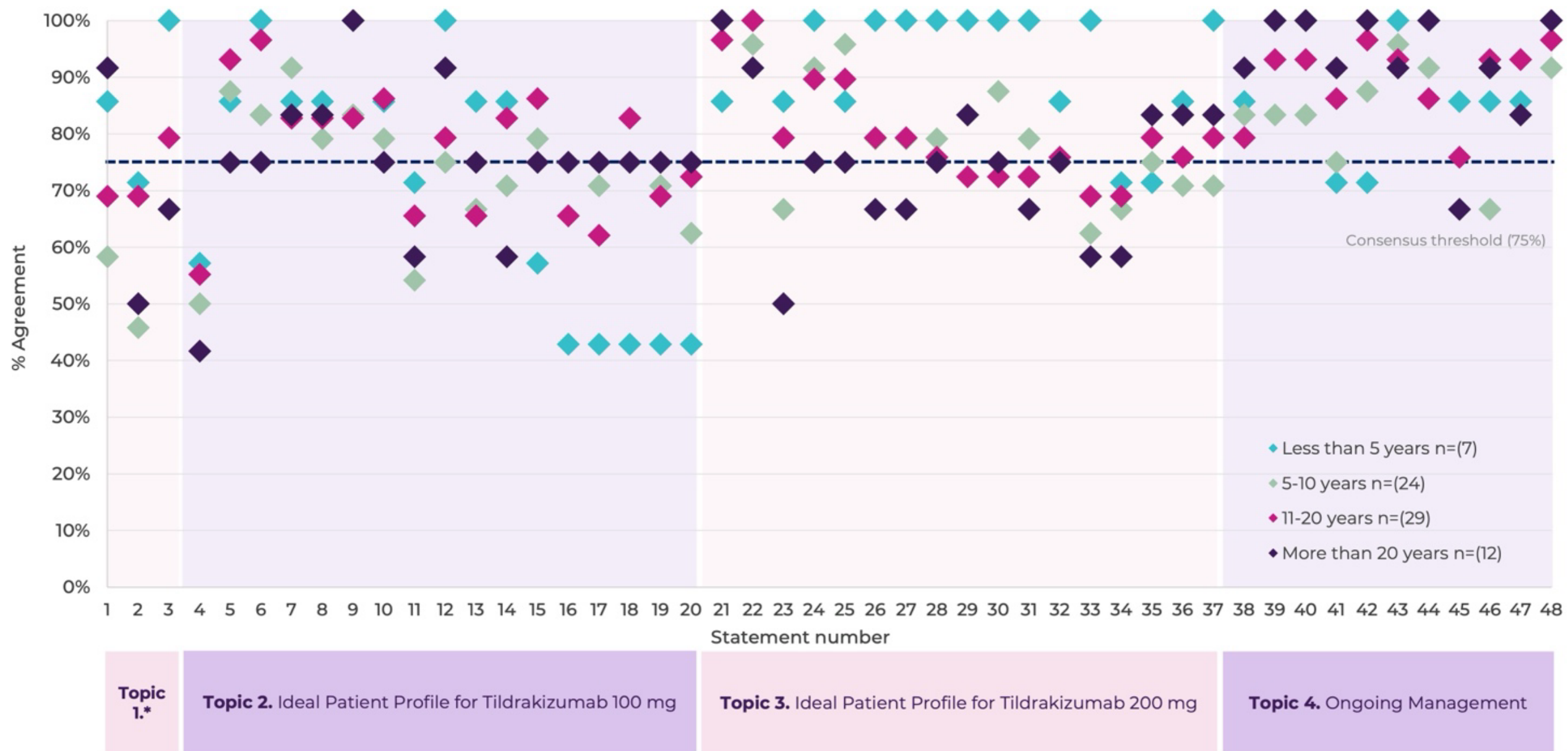

Figure S6. Sub-analysis of responses by years of experience in role. *Variations in current practice.

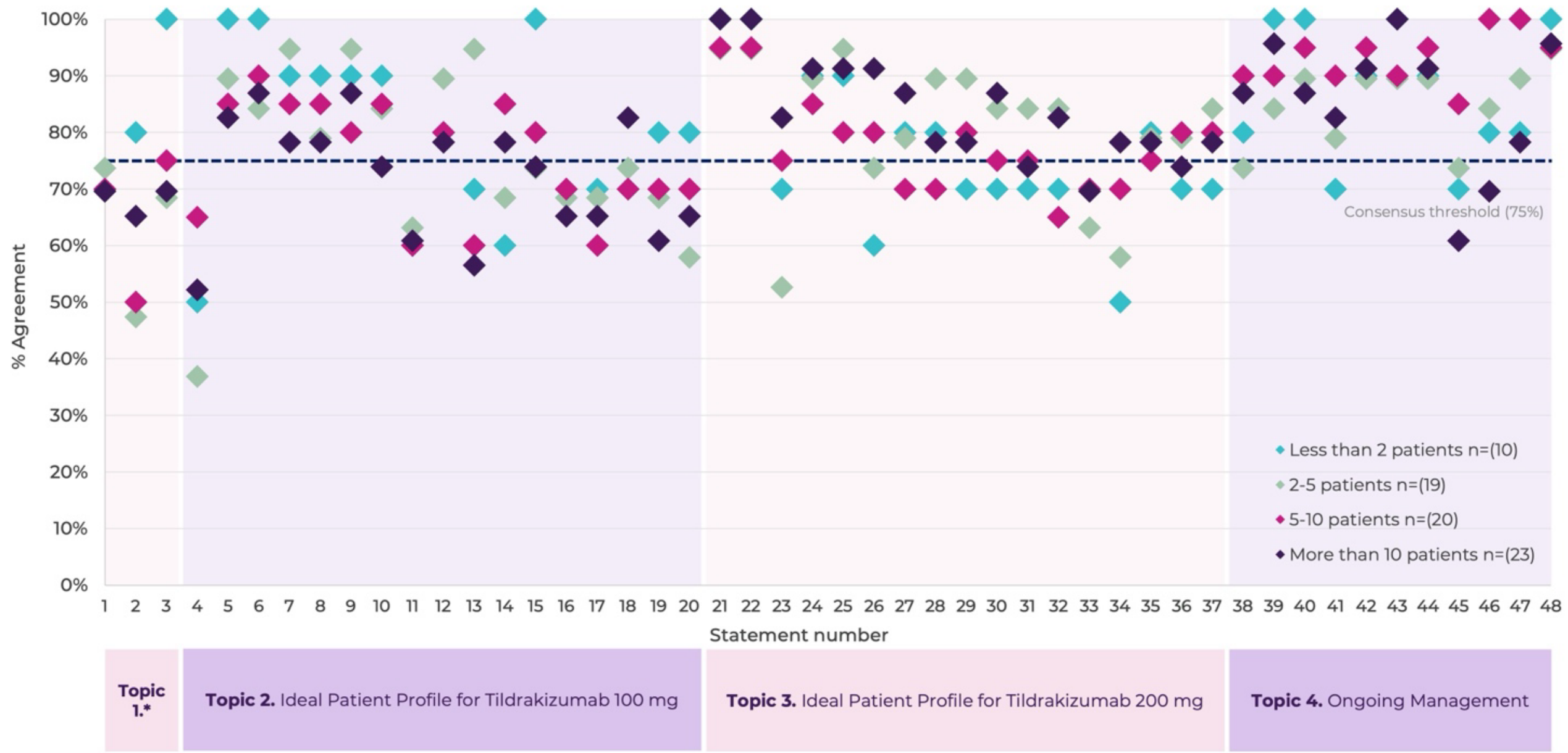

Figure S7. Sub-analysis of responses by number of patients treated with tildrakizumab in the last year. *Variations in current practice.

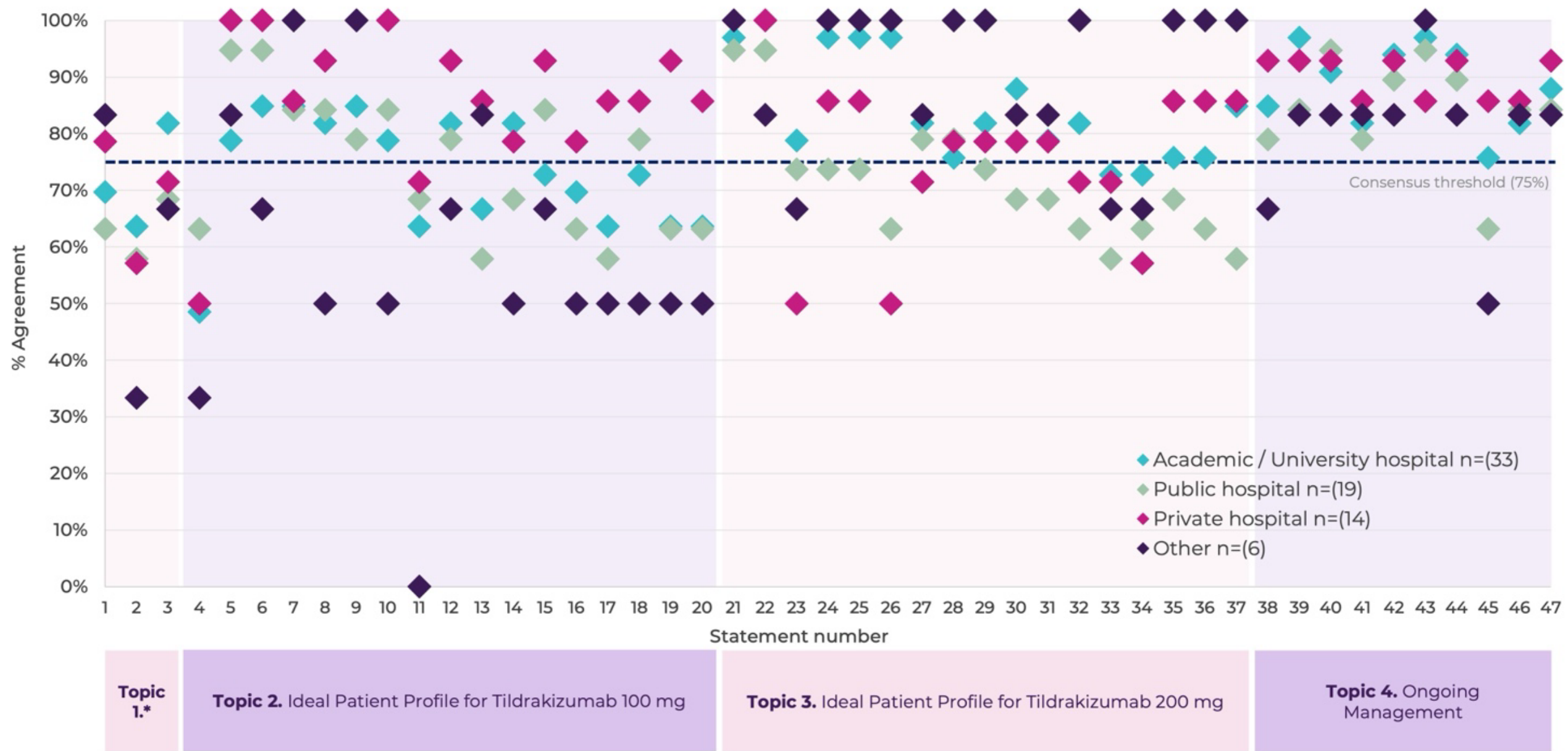

Figure S8. Sub-analysis of responses by primary institution. *Variations in current practice.
